# Surgical Strategies in Renal Cancer: A Meta-analysis of Partial vs. Radical Nephrectomy Outcomes Across Tumor Stages

**DOI:** 10.5339/qmj.2025.54

**Published:** 2025-06-09

**Authors:** Ahmad R. Al-Qudimat, Seif B. Altahtamouni, Mai Elaarag, Kalpana Singh, Meiad Abdelrahman, Ibrahim A. Khalil, Samer A. Hasan, Islam Al-Oweidat, Omar M. Aboumarzouk

**Affiliations:** 1 Surgical Research Section, Department of Surgery, Hamad Medical Corporation, Doha, Qatar; 2 Department of Public Health, College of Health Sciences, QU-Health, Qatar University, Doha, Qatar; 3 Department of Business Intelligence Unit, Hamad Medical Corporation, Doha, Qatar; 4 Department of Nursing, Hamad Medical Corporation, Doha, Qatar; 5 Department of Urology, Hamad Medical Corporation, Doha, Qatar; 6 Department of Community and Mental Health Nursing, College of Nursing Sciences, Zarqa University, Zarqa, Jordan; 7 College of Medicine, QU-Health, Qatar University, Doha, Qatar; 8 School of Medicine, Dentistry and Nursing, The University of Glasgow, Glasgow, UK *Email: aalqudimat@hamad.qa

**Keywords:** Kidney neoplasms, renal cell carcinoma, partial nephrectomy, radical nephrectomy, nephrectomy, treatment outcome, postoperative complications, mortality, neoplasm staging

## Abstract

**Background:**

Surgical intervention remains the primary treatment for localized renal tumors and masses, with partial nephrectomy (PN) and radical nephrectomy (RN) being the two most frequently employed procedures. The choice between these approaches is often influenced by factors such as tumor size, location, histology, and patient comorbidities. However, the decision between PN and RN remains a subject of ongoing debate, particularly as emerging evidence suggests varying outcomes based on the stage and type of renal tumors. This meta-analysis evaluates the association between renal tumor stage and subtype with the outcomes of PN and RN, focusing on renal function, cancer-specific survival, and postoperative complications.

**Method:**

An exhaustive search was conducted across PubMed, Scopus, and Embase databases, covering the literature from their inception up to March 2023, in accordance with the Preferred Reporting Items for Systematic reviews and Meta-Analyses (PRISMA) guidelines. Original studies comparing PN to RN in the management of renal tumors at various stages were meticulously screened, adhering to stringent inclusion and exclusion criteria. This protocol was registered on PROSPERO (CRD42023455985).

**Result:**

Overall, 38 cohort studies were included, with a total of 144,608 patients diagnosed with renal cancer who underwent nephrectomy, 71,582 who underwent PN, and 72,671 who underwent RN. The data revealed a significant difference in cancer-specific survival between PN and RN, which was higher in the RN group (pooled HR: 1.17; 95% CI = 1.01–1.35) *p* < 0.001. The postoperative renal function of patients who underwent RN was worse than that of patients who underwent PN (pooled RR: 4.22; 95% CI: 1.45, 12.27, *p* < 0.00001). The relative risk of papillary renal cell carcinoma (RCC) was lower in patients who underwent RN as compared to PN (the pooled RR, 1.32; 95% CI = 1.02, 1.72, *p* < 0.001), while the relative risk of RCC collecting duct subtype was significantly lower patients who underwent PN as compared to RN (the pooled RR, 0.44 (95% CI = 0.29, 0.67) *p* = 0.97. Additionally, the pooled risk for patients with a Charlson Comorbidity Index score of ≥2 was lower in the PN group compared to the RN group.

**Conclusion:**

Across various tumor stages, RN demonstrates superior cancer-specific survival, and a lower incidence of postoperative complications compared to PN. However, PN is associated with more favorable renal function preservation. These findings, in conjunction with individual patient characteristics, should be meticulously evaluated to inform the selection of the most appropriate surgical approach and guide patient counseling.

## Introduction

Renal cell carcinoma (RCC), originating in the renal cortex, is the most common primary renal neoplasm, representing 80%–85% of all primary renal cancers. Transitional cell carcinoma and other rare cancers that arise in the renal pelvis constitute the remaining 15%–20%.^1^

In 2020, RCC accounted for 2.2% of all cancer cases, with over 431,288 new cases, and 179,368 deaths. Age-standardized incidence and mortality rates of kidney cancer were higher in developed countries such as North America, Europe, and Australia. Men have a higher incidence of kidney cancer than women.^2,3^

The European Association of Urology recommends surgical interventions as the benchmark for the treatment of localized RCC. The two primary surgical options in managing RCC are radical nephrectomy (RN), which is the removal of the entire affected kidney, and partial nephrectomy (PN), which is the removal of only the tumor itself and a part of the healthy kidney tissue.^4^ The stage of cancer and the need for preservation of renal function are among the most important factors that contribute to the choice of one surgical procedure over another. For example, PN is preferred in cases of early-stage kidney cancer in order to preserve renal function and lower the risk of complications.^5^

Classification of renal masses according to the Tumor, Nodes, Metastasis (TNM) system is recommended for clinical and scientific staging. The prognostic value of the TNM classification has been validated in both single- and multi-institution studies.^4^ Nevertheless, the clinical guidelines recommend that PN be performed on patients with clinical stage T1 renal cell tumors (≤7 cm). PN has been shown to have a similar oncological outcome and overall survival to RN.^6,7^ Moreover, PN preserves better postoperative renal function and, therefore, lowers the risk of cardiovascular and metabolic sequelae. Additionally, PN has an acceptable perioperative complications profile.^7–10^

RN is still the benchmark for treating patients with T2 renal cell tumors and localized tumors when not amenable to be treated by PN. Tumor upstaging can be the result of high tumor complexity, increasing tumor diameter, and hilar location, which in turn warrants the use of RN rather than nephron-sparing treatment like PN.^11^

The role of PN in stage T2 tumors is controversial, as it is not clear whether it would give better or similar results to RN. Recent studies are trying to investigate if the potential benefits outweigh the risks of PN for patients with T2 tumors. PN is beneficial for patients with T2 renal tumors in terms of overall survival and the protection of renal function. However, perioperative complications should be considered when facing larger T2 tumors.^9,10^ The use of PN for the treatment of RCC has become more popular in recent years.^12^ The use of new approaches, such as robot-assisted PN, has minimized the technical challenges faced with performing conventional minimally invasive procedures and reduced the rate of complications.^13^

This meta-analysis was conducted to compare the use of PN and RN for the treatment of RCC across various tumor stages in terms of cancer-specific survival, all-cause mortality, functional outcomes, and postoperative complications.

## Method

This meta-analysis was performed following the Preferred Reporting Items for Systematic reviews and Meta-Analyses (PRISMA) guidelines.^14^

### Search strategy

A thorough search was conducted in PubMed, Embase, and Scopus databases from inception until March 2023, focusing on published prospective, retrospective, cross-sectional, case-control, and preprint papers, that have been accepted for publication and were also taken into consideration.

The search included keywords and medical subject headings (MeSH), such as “partial nephrectomy” OR “nephron-sparing surgery” OR “radical nephrectomy” OR “total nephrectomy” OR “Nephrectomy/classification” [MeSH] OR “Nephrectomy/instrumentation” [MeSH] OR “Nephrectomy/methods” [MeSH]) for the treatment terms, and Renal cell carcinoma OR renal tumors OR (“Kidney Neoplasms/complications” [MeSH] OR “Kidney Neoplasms/history” [MeSH] OR “Kidney Neoplasms/mortality” [MeSH] OR “Kidney Neoplasms/surgery” [MeSH]) for the diagnostic terms.

Initial search yielded a total of 12,135 articles. Title and abstract screening were performed on 5,692 articles after the removal of duplicates, non-English articles, nonhuman articles, editorials, case reports, comments, and reviews. A total of 258 studies were eligible for full-text screening, of which 38 articles were selected for this systematic review and meta-analysis.

### Criteria and study selection

The following criteria were used to determine which studies will be included in this review: (a) adult patients greater than 18 years, (b) PN vs. RN for the treatment of renal tumors, (c) with no restrictions on tumor stage or grade, (d) studies reporting mortality rates in these patients either prospective or retrospective, (e) English report publication. These were the exclusion criteria: (a) duplicate reports (including same patients’ information), (b) insufficient data, (c) reviews and reports, (d) the treatment was done by a modality other than PN or RN, (e) the study evaluated one procedure with no comparison group, and (f) if the study was a case report, review, comment, editorial, or concerned with animal studies.

Two authors independently screened and extracted the articles by title and abstract and then by full text to decide on the included studies. Any differences between reviewers at each stage of the screening process were discussed with senior authors, and a consensus decision for eligibility was made.

### Data extraction

Three authors extracted variables from the information provided (first name of the author, study design, sample size, country, etc.) and according to the main stratification variable (cancer-specific mortality, cancer-specific survival, all-cause mortality, etc.).

### Statistical analysis

The meta-analysis employed log hazard ratio (HR) and variance as the summary outcome measures across all studies. We calculated the HR at the 95% confidence interval (CI) for all-cause mortality, cancer-specific survival, and overall survival. The risk ratio was used as the summary outcome measure from all studies in the meta-analysis. We calculated the RR with a 95% CI of the data for each study to compare PN with RN. Also, the risk ratio (RR) with 95% CI was used to compare the papillary RCC, RCC collecting duct type, cancer grade, and subtype of clear cell renal carcinoma patients with PN or RN group. To ascertain the summary RR’s statistical significance, the Z-test was used. To assess the degree of heterogeneity between the studies, the *I*^2^-test and chi-square test were used. The *I*^2^ value was used to determine the degree of heterogeneity (*I*^2^ = 25%, no heterogeneity; *I*^2^ = 25–50%, moderate heterogeneity; *I*^2^ > 50%, high or extreme heterogeneity). The random-effects (DerSimonian-Laird method) models were employed to evaluate the pooled RR to test the results’ reliability. Funnel plots with the Egger regression test were used to assess publication bias.^15^ STATA 17.0 was used for all the analyses; *p*-values that were <0.05 were considered statistically significant. All statistical tests were 2-sided.

### Protocol registration

The protocol for this systematic review was registered in the International Prospective Register of Systematic Reviews (PROSPERO) with a unique number (CRD42023455985).

### Quality assessment

Newcastle-Ottawa Scale was used for the quality appraisal for each article; the risk of bias assessment was performed separately for each study.^16^ This tool comprises three components and a concluding line. Items one through four evaluate the selection and comparability of items, with the final item appraising the outcome. The Newcastle-Ottawa Scale yields ratings indicating poor quality (0 or 1), fair quality (2), and good quality (up to 3).

## Results

### Search findings flow chart

**Figure 1:**
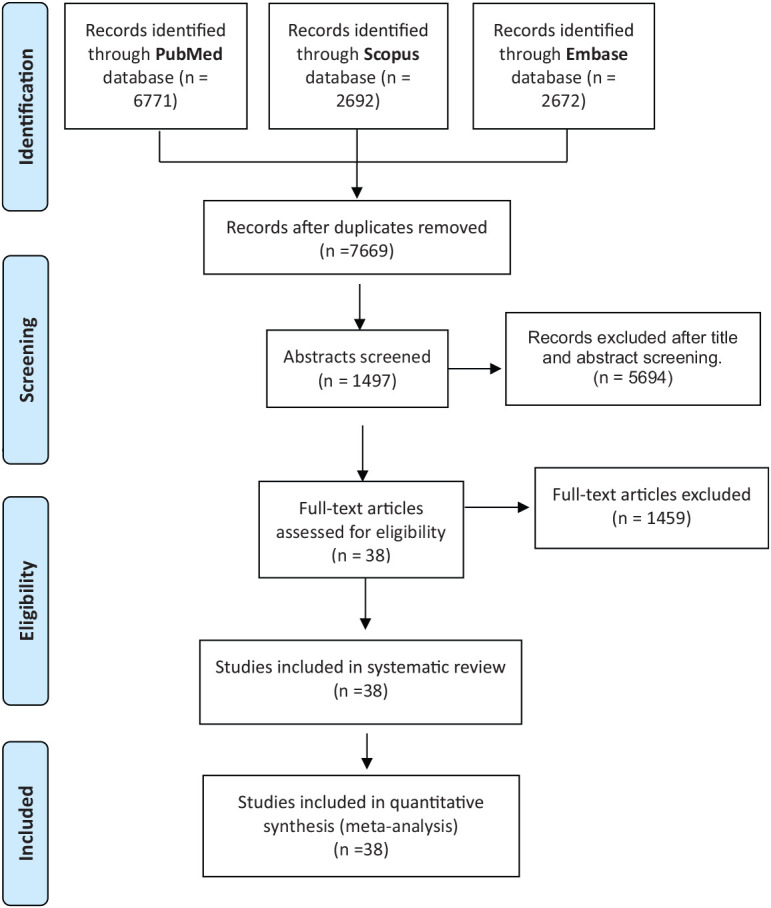
Flow chart of included studies.

The literature review yielded a cumulative total of 12,135 studies, drawing data from various databases, including PubMed (N = 6771), Scopus (N = 2692), and Embase (N = 2672). Among these, 4,466 studies were excluded due to duplication, while an additional 4,428 studies did not meet the inclusion criteria, specifically due to incorrect exposure, outcome, and other reasons detailed in Figure 1. Consequently, a total of 38 studies were deemed suitable for inclusion in the systematic review and meta-analysis.

### Study characteristics

In this review, we have included 38 studies, including 144,608 patients who had nephrectomy (PN, 71582; RN, 72671). The studies were published from 1970 to 2020. Thirty-eight studies; 35 were retrospectives, and three were prospective. Most included studies from the USA, then Europe and Asia (Table 1). Thirteen studies reported the overall survival, five studies reported all-cause mortality, and six studies reported cancer-specific mortality and cancer-specific survival. The median score of the study was 7.13 (range: 6–9). Of 38 studies, 23 were rated to have good quality, and the remaining 15 were of fair quality with 0 poor quality (Table 1).

**Figure 2:**
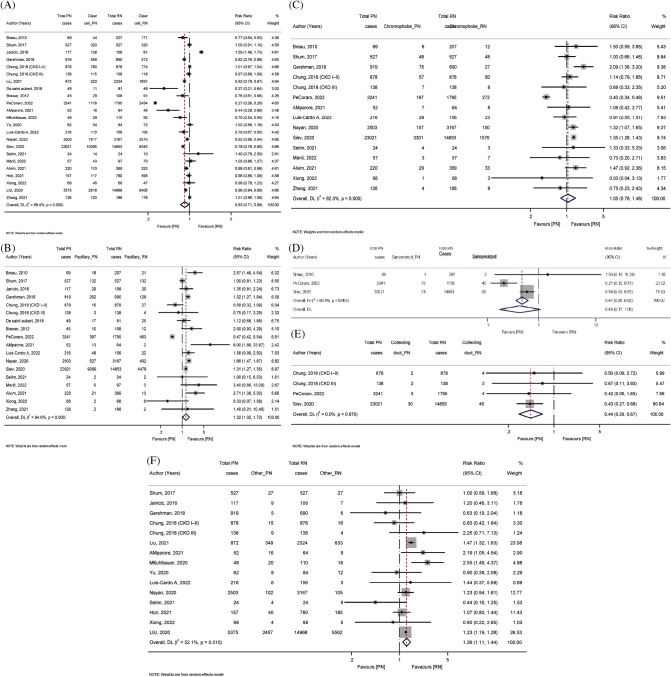
Forest plot of (A) histological subtypes of clear cell renal carcinoma, (B) the papillary carcinoma, (C) RCC chromophobe type, (D) RCC sarcomatoid subtype, (E) RCC collecting duct type, and (F) RCC other type.

Twenty-three studies reported the histological subtypes of clear cell renal carcinoma. The heterogeneity among the studies is obvious, indicated by an *I*^2^ value of 99.3% and a *p*-value of <0.001. There was a statistically significant difference between the PN and the RN group (RR: 0.83, 95% CI: 0.71–0.98, *p* = 0.026, *z* = −2.227, random effect model (Figure 2A). Eighteen studies reported papillary RCC. The pooled relative risk was calculated using the random effect model to take the heterogeneity into account. The data revealed that papillary carcinoma had a lower risk in patients who underwent RN as compared to PN (the pooled RR, 1.32 (95% CI: 1.02, 1.72), Z = 2.076, *p* = 0.038)) with *I*^2^ = 94.6%, Q = 316.13, *p* < 0.001, Figure 2B. Four studies reported that in the RCC collecting duct type, there was a significantly lower risk in favor of PN as compared to RN (the pooled RR, 0.44 (95% CI: 0.29, 0.67), Z = −3.842, *p* < 0.001)) with *I*^2^ = 0.0%, Q = 0.25, *p* = 0.97 (Figure 2E). Fifteen studies reported RCC of other types. There was a significantly higher risk in the PN group as compared to the RN group. The pooled RR, 1.26 (95% CI: 1.11, 1.44), Z = 3.496, *p* < 0.001)) with a moderate level of heterogeneity *I*^2^ = 52.2%, Q = 29.21, *p* = 0.01 (Figure 2F). The data showed that the PN group had fewer papillary carcinoma cases and fewer RCC collecting duct type cases. There was no significant difference between the PN and RN groups in terms of RCC chromophobe type and RCC sarcomatoid subtype (Figures 2C and D).

**Figure 3:**
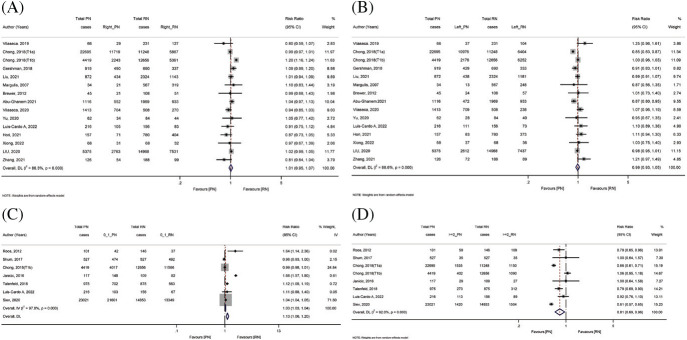
Forest plot comparing tumor side and Charlson Comorbidity Score (CCS) in patients receiving PN vs. RM. (A) Tumor side right, (B) tumor side left, (C) CCS = 0–1, and (D) CCS ≥ 2.

Fifteen studies reported the tumor side (left as well as right). We did not find any statistical significance with the tumor side, i.e., left and right with PN as well as RN (Figures 3A and B).

Seven studies reported the Charlson comorbidity index with a 0–1 score. A random effect model revealed that the pooled risk was significantly in favor of RN group (pooled RR = 1.13, 95% CI: 1.06–1.20, Z = 3.889, *p* < 0.001). The studies had a higher amount of heterogeneity (*I*^2^ = 92%, Q = 277.99, *p* ≤ 0.001) (Figure 3C).

Eight studies reported the Charlson Comorbidity Index with a ≥2 score. The pooled risk was lower in the PN group as compared to the RN group (pooled RR = 0.81, 95% CI: 0.69–0.96, Z = −2.418, *p* < 0.016). The studies had a higher amount of heterogeneity (*I*^2^ = 97.8%, Q = 87.76, *p* ≤ 0.001) (Figure 3D).

**Figure 4:**
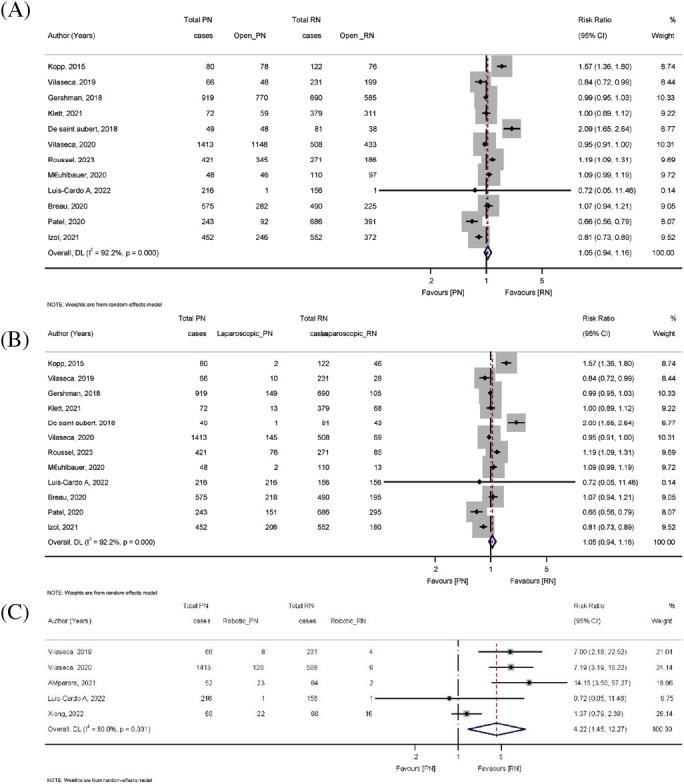
Forest plot comparing surgery types—(A) open, (B) laparoscopic, and (C) robotic surgery—in patients receiving PN vs. RN.

There was no significant difference between the PN and RN groups in terms of open and laparoscopic surgery (Figures 4A and B). Moreover, five studies reported the robotic surgery type. The postoperative renal function of patients who underwent RN was worse than that of patients who underwent PN (pooled RR: 4.22; 95% CI: 1.45, 12.27; *p* < 0.00001) (Figure 4C); thus, it seems that PN is superior in preserving renal function postoperatively.

**Figure 5:**
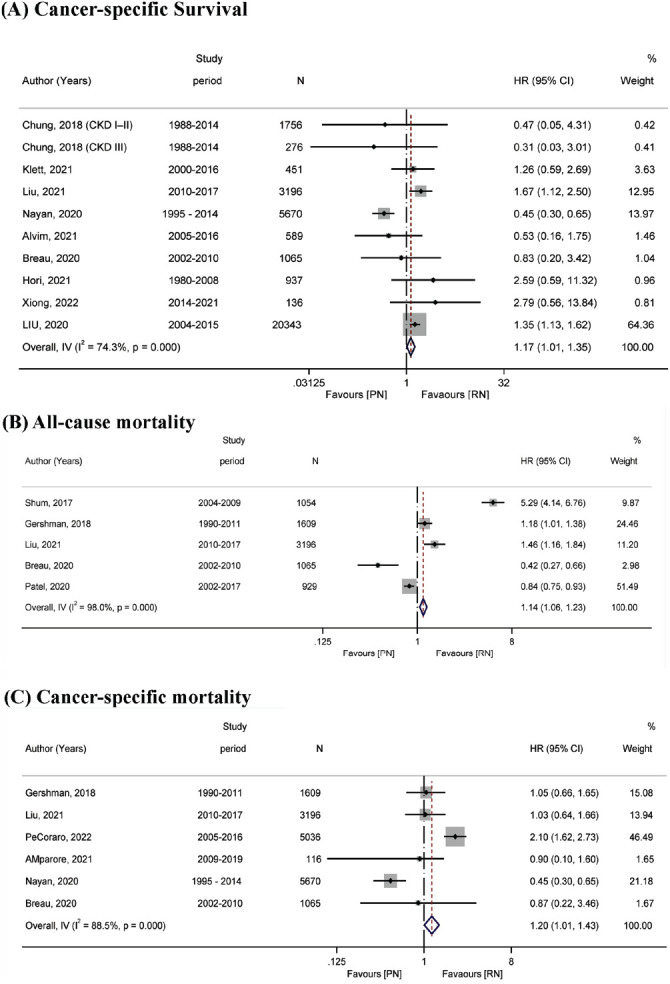
Forest plot comparing surgery types—(A) cancer-specific survival, (B) all-cause mortality, and (C) cancer-specific mortality—in patients receiving PN vs. RN.

In terms of long-term outcomes, cancer-specific survival was higher in the RN group, indicating that PN and RN were significantly different (pooled HR: 1.17; 95% CI: 1.01–1.35) with higher heterogeneity *I*^2^ = 74.3%, *p* < 0.001 (Figure 5A).

Five studies reported all-cause mortality, and the combined results showed a clear difference between PN and RN in terms of all-cause mortality (HR: 1.14; 95% CI: 1.06, 1.23; *p* = 0.001) (Figure 5B). Patients who underwent PN generally had higher mortality than those who underwent RN, with higher heterogeneity (*I*^2^ = 98%). The combined effect size of cancer-specific mortality of PN patients was higher than that of RN patients (HR: 1.20; 95% CI: 1.01, 1.43; *p* = 0.04 (Figure 5C) with higher heterogeneity *I*^2^ =88.5%, Q = 43.49, *p* < 0.001).

## Discussion

The aim of our research was to conduct a thorough and comprehensive systematic review and meta-analysis to determine the better surgical choice between PN and RN in the treatment of renal tumors in terms of oncological outcomes. Our analysis was done for the sole purpose of helping surgeons make a choice between the two treatment modalities.

According to the literature, RN predisposes to chronic kidney disease (CKD), which can lead to comorbid cardiovascular events and increased mortality, while PN is associated with increased surgical urological complications such as urine leak and postoperative bleeding.^54^ Both should be taken into consideration in the management of RCC. In a prospective, randomized study in NSS or RN conducted by the European Organization for Research and Treatment of Cancer Genito-Urinary Group (EORTC-GU) noninferiority phase 3 trial 30904, it was found that in PN vs. RN, the incidence of bleeding (3.1% vs. 1.2%), urinary fistula (4.4% vs. 0%) and the rate of secondary operations (4.4% vs. 2.4%) were higher in the PN group.^55^ This was proven in a study conducted by Huang, Ruizhen et al., which showed that PN required a longer operation time (MD: 44.85 minutes; 95% CI: 8.17, 81.52; *p* = 0.02) and higher intraoperative blood loss than RN (MD: 103.85 mL; 95% CI: 77.13, 103.57; *p* < 0.00001).^10^

Two retrospective studies by Kopp et al.^17,56^ examined the survival rate and renal function score of PN vs. RN, it revealed that both surgical methods had no significant difference in terms of renal function score and survival rate of patients with T2 tumors, but the incidence of complications in the PN group was significantly higher than that in the RN group (17.5% vs. 2.5%; *p* < 0.001), and 10% of patients had urine leakage after surgery, it also reported longer operation time in PN vs. RN (221 minutes vs. 153 minutes, *p* = 0.001), longer intraoperative exposure leads to increased incidence of postoperative complications and with larger more complex tumors, its resection via PN can reduce the benefits of renal function and NSS.

A retrospective study conducted by Bigot, Pierre et al.^57^ evaluating the morbidity, functional, and oncological outcomes after NSS for renal tumors >7 cm reported that NSS for larger tumors is associated with significant complication and renal function deterioration rates. However, a study by Alanee et al. using the Surveillance, Epidemiology, and End Results (SEER) database to analyze surgical treatment of T2 tumors showed that the cancer-specific mortality was not inferior for patients treated with PN versus RN (HR 0.68, 95% CI 0.50–0.94) and PN for T2 renal masses has no contraindicated effect on CSS.^58^ Breau et al.^59^ studied PN vs. RN in the treatment of clinically staged T2 renal tumors retrospectively and reported a low complication rate in both groups and that urine leak in the PN group was resolved spontaneously under the action of the drainage tube and/or ureteral stent placed after the abdominal cavity was closed, therefore, concluded the safety and effectiveness of PN in the treatment of renal cancer regardless of tumor stage.

In a meta-analysis conducted by Huang, Ruizhen et al.^10^ revealed no obvious difference in cancer-specific mortality and cancer-specific survival, which indicates that PN and RN are not significantly different in the above two aspects (HR: 0.91; 95% CI: 0.68, 1.21; *p* = 0.66). Our combined meta-analysis, however, showed that in terms of long-term outcome and cancer-specific survival, the difference between PN and RN was statistically significant (pooled HR: 1.17; 95% CI: 1.01–1.35, *p* < 0.001) in favor of RN and cancer-specific mortality of RN patients was higher than that of PN patients (HR: 1.20; 95% CI: 1.01, 1.43; *p* = 0.04). Pecoraro et al.,^34^ using the SEER database, studied patients with stage T3a RCC undergoing RN vs. PN and revealed a twofold increase in CSM rate after RN vs. PN. In patients with stage T1a RCC, however, a study performed by Gershman et al.^25^ showed no statistically significant difference in CSM and ACM in RN vs. PN in the treatment of stage T1a RCC. In analyzing all-cause mortality, the combined results showed that patients who underwent RN had a higher mortality rate than those who underwent PN (HR: 1.14; 95% CI: 1.06, 1.23; *p* = 0.001) with higher heterogeneity *I*^2^ = 88.5%, Q = 43.49, *p* < 0.001. This was also proven by a previous study mentioned,^10^ which concluded that patients who underwent PN generally had a longer OS than those who underwent RN. The literature demonstrates equivalent oncologic outcomes for PN compared with RN in appropriately selected patients, and the functional outcomes tilt the balance in favor of PN whenever feasible.^54^

RCC is conventionally assessed according to the Union for International Cancer Control (UICC) and the American Joint Committee on Cancer (AJCC) recommendations, which categorize the major RCC histologic subtypes as clear cell, papillary, chromophobe, cystic, collecting duct, and medullary.^60^ Clear cells comprise 70%–80% of renal cell cancers, followed by papillary with 10%–15%, and chromophobe at 3%–5%. According to the literature, important prognostic factors for cancer-specific survival in patients with nonmetastatic RCC include specific clinical signs or symptoms, tumor-related factors, and various laboratory findings. Overall, tumor-related factors such as pathologic stage (most important), tumor size, nuclear grade, and histologic subtype have the greatest individual predictive ability.^10^ In a study conducted by Siev et al.^60^ to evaluate OS of T1a kidney cancer stratified by histological subtypes and treatment modality, it concluded that OS was worse for RN than PN for clear cell (HR 1.38 [1.28–1.50]), papillary (1.34 [1.16–1.56]), and chromophobe RCC (1.92 [1.43–2.58]). At the same time, the effect of treatment modality on OS was not observed with sarcomatoid or collecting duct tumors.^43^ This goes with our combined meta-analysis that showed that there was no significant difference in OS between the PN and RN groups in terms of RCC chromophobe type and RCC sarcomatoid subtype, which may be due to the presentation of sarcomatoid and collecting duct tumor variants which are more likely to present with advanced disease, infrequently found to be <4 cm at the time of diagnosis.^61^ Our study showed a significantly lower risk in favor of PN as compared to RN in RCC collecting duct type (RR 0.44 (95% CI: 0.29,0.67. *p* < 0.001), and in papillary cell carcinoma, the risk was lower in patients who underwent RN compared to those who underwent PN with an RR of 1.32 (95% CI: 1.02, 1.72. *p* = 0.038).

Trends in renal surgery have changed over the past years, and advancements in technology have been evident across various fields. Experience with laparoscopic PN using pure and robot-assisted approaches continues to increase with an increasing number of centers performing these surgeries for gradually expanding indications.^62^ Five of our studies included in this meta-analysis reported robotic surgery, with it being more popular in partial nephrectomies rather than radical. In comparison with a previous meta-analysis reported in 2017 by Xia et al., their results showed that robot-assisted PN is associated with fewer postoperative complications (including major and minor complications), comparing the need for blood transfusion, estimated blood loss, and length of hospital stay.^63^

Our meta-analysis, however, showed better outcomes with robotic RN in controlling postoperative complications (pooled RR 4.22, 1.45, 12.27, *p* = 0.001) and found no significant difference between PN and RN in terms of open and laparoscopic surgeries. In a study published by Lane et al.^64^ comparing laparoscopic and open techniques in PN, it concluded that the use of either approach should be determined based on the features of the individual patient and tumor and the expertise of the surgeon.

### Limitations

Our study contained some limitations. Firstly, all included studies were observational, with a significant difference in sample sizes, which in turn introduced a degree of bias and heterogeneity. Secondly, due to insufficient data, we could not examine various factors that can alter the choice of surgery, including learning curves and experiences among surgeons. Lastly, the span and follow-up periods between studies varied significantly, during which surgical guidelines and indications may have been changed.

### Strengths

This study provides a comprehensive comparison of PN and RN across all stages of renal tumors, highlighting the nuanced differences in oncological, perioperative, and long-term outcomes between these two surgical approaches. The use of a large sample size enhances the reliability and generalizability of the findings, offering valuable insights into the relative benefits and risks of each treatment modality. Additionally, the study emphasizes the importance of individualized treatment planning, considering patient-specific factors and tumor characteristics, which is critical for optimizing surgical outcomes. The inclusion of both perioperative and oncological outcomes provides a well-rounded analysis, contributing to a more informed and evidence-based approach to clinical decision-making in renal cancer management.

## Conclusion

With the increasing use of PN for renal tumors across all stages, it is evident that PN offers superior perioperative outcomes and better preservation of kidney function. However, PN may carry a higher risk of cancer recurrence compared to RN, which is, on the other hand, associated with worse long-term effects on kidney function. A comprehensive assessment of individual patient factors, including tumor characteristics and renal function, is essential to design a patient-centered treatment plan and counseling. Further large-scale, prospective studies with larger sample sizes are needed to refine surgical decision-making, particularly for patients with more complex renal tumors, ensuring that the choice between PN and RN is optimized for each patient’s unique clinical situation.

## Conflicts of interest

None declared.

## Authors’ contribution

ARA-Q: conceptualization, methodology, supervision, writing—review and editing; SBA: project administration, writing—review; ME: project administration, data collection, writing—review; KS: project administration, analysis, writing—review and editing; MA: project administration, data collection, writing—review; IK: project administration, data collection, writing—review; SH: project administration, data collection, writing—review; OMA: conceptualization, methodology, supervision, writing—review and editing.

## Acknowledgments

The publication of this article was funded by the Qatar National Library (QNL).

## Figures and Tables

**Table 1.** Provides a summary of the features of the included studies.

**Author years**	**Study design**	**No. of patients**		**Country**	**Study period**	**NOS**
		**PN**	**RN**			
Kopp R, et al.^17^	Retrospective	80	122	USA	2002–2012	7 (S:3, C:1, O:3)
Vilaseca A., et al.^18^	Retrospective	66	231	USA	2000–2012	6 (S:3, C:0, O:2)
Breau Rh., et al.^19^	Retrospective	69	207	USA	1970–2008	8 (S:3, C:2, O:3)
Roos CR, et al.^20^	Retrospective	101	146	Germany	1988–2007	8 (S:3, C:2, O:3)
Shum CF, et al.^21^	Retrospective	527	527	USA	2004–2009	8 (S:3, C:2, O:3)
Chong JT, et al.^22^ (T1a)	Retrospective	22,695	11,248	USA	2004–2013	9 (S:4, C:2, O:3)
Chong JT, et al.^22^ (T1b)	Retrospective	4,419	12,656	USA	2004–2013	9 (S:4, C:2, O:3)
Janicic A, et al.^23^	Prospective	117	109	Serbia	1996–2013	6 (S:3, C:0, O:3)
Talenfeld AD, et al.^24^	Retrospective	975	875	USA	2006–2013	6 (S:3, C:0, O:3)
Gershman B, et al.^25^	Retrospective	919	690	USA	1990–2011	8 (S:3, C:2, O:3)
Chung J, et al.^26^ (CKD I–II)	Retrospective	878	878	Korea	1988–2014	8 (S:3, C:2, O:3)
Chung J, et al.^26^ (CKD III)	Retrospective	138	138	Korea	1988–2014	8 (S:3, C:2, O:3)
Klett DE, et al.^27^	Retrospective	72	379	USA	2000–2016	8 (S:3, C:2, O:3)
Liu S, et al.^28^	Retrospective	872	2324	China	2010–2017	7 (S:3, C:1, O:3)
Margulis V, et al.^29^	Retrospective	34	567	USA	1990–2006	7 (S:3, C:1, O:3)
Aubert N, et al.^30^	Retrospective	49	81	France	2000–2013	6 (S:3, C:0, O:3)
Brewer K, et al.^31^	Retrospective	45	108	USA	2004–2010	7 (S:3, C:1, O:3)
Janssen M, et al.^32^	Retrospective	18	105	Germany	1980–2010	6 (S:3, C:0, O:3)
Abu–Ghanem Y, et al.^33^	Retrospective	1116	1969	EU-10	2006–2011	7 (S:4, C:0, O:3)
PeCoraro A, et al.^34^	Retrospective	1795	11.382	Multi	2005–2016	8 (S:3, C:2, O:3)
Vilaseca A, et al.^18^	Retrospective	1413	508	multi	2000–2012	7 (S:3, C:1, O:3)
AMparore D, et al.^35^	Retrospective	52	64	Italy	2009–2019	6 (S:3, C:0, O:3)
Roussel E, et al.^36^	Retrospective	421	271	USA	2003–2019	6 (S:3, C:0, O:3)
Kim H, et al.^37^	Retrospective	88	44	Korea	2003–2019	8 (S:3, C:2, O:3)
Lam JKL, et al.^38^	Retrospective	30	37	Singapore	2005–2014	7 (S:3, C:1, O:3)
M€uhlbauer J, et al.^39^	Retrospective	48	110	Germany	2005–2019	7 (S:3, C:1, O:3)
Yu K, et al.^40^	Retrospective	62	84	China	Ns	8 (S:3, C:2, O:3)
Luis-Cardo A, et al.^41^	Retrospective	216	156	Spain	2005–2018	6 (S:3, C:0, O:3)
Nayan M, et al.^42^	Retrospective	2,503	3,167	Canada	1995–2014	7 (S:4, C:0, O:3)
Siev M, et al.^43^	Retrospective	23021	14853	USA	2004–2015	7 (S:4, C:0, O:3)
Amer M, et al.^44^	Prospective	24	24	Egypt	2018–2020	6 (S:3, C:0, O:3)
Marić P, et al.^45^	Prospective	57	97	Serbia	2006–2013	6 (S:3, C:0, O:3)
Alvim R, et al.^46^	Retrospective	220	369	USA	2005–2016	7 (S:3, C:0, O:3)
Breau RH, et al.^47^	Retrospective	575	490	Canada	2002–2010	8 (S:4, C:1, O:3)
Hori S, et al.^48^	Retrospective	157	780	Japan	1980–2008	7 (S:3, C:1, O:3)
Xiong S, et al.^49^	Retrospective	68	68	China	2014–2021	8 (S:3, C:2, O:3)
Patel SH, et al.^50^	Retrospective	243	686	USA	2002–2017	8 (S:3, C:2, O:3)
Izol V, et al.^51^	Retrospective	452	552	Turkey	2000–2018	7 (S:4, C:0, O:3)
Liu X, et al.^52^	Retrospective	5,375	14,968	China	2004–2015	7 (S:3, C:2, O:2)
Zhang X, et al.^53^	Retrospective	126	188	China	2013–2018	8 (S:3, C:2, O:3)

NOS: Newcastle-Ottawa Scale, Ns: not stated.
